# Symptomatic Recurrent Chylopericardium: How to Manage This Rare Entity?

**DOI:** 10.1016/j.jaccas.2021.06.011

**Published:** 2021-08-19

**Authors:** Ankit Agrawal, Beni R. Verma, Jeremy Brooksbank, Mohamed Khayata, Allan L. Klein

**Affiliations:** a Center for the Diagnosis and Treatment of Pericardial Diseases, Section of Cardiovascular Imaging, Department of Cardiovascular Medicine, Heart, Vascular, and Thoracic Institute, Cleveland Clinic, Cleveland, Ohio, USA; b Department of Cardiovascular Medicine, University of South Florida Morsani College of Medicine, Tampa, Florida, USA

**Keywords:** echocardiography, pericardial effusion, post-operative, CPE, chylopericardium, CT, computed tomography, JVP, jugular venous pulse, CMR, cardiac magnetic resonance, PE, pericardial effusion, TTE, transthoracic echocardiogram

## Abstract

Recurrent chylopericardium after cardiac surgery is a rare entity. This paper presents the case of a 69-year-old female who developed a large recurrent chylopericardium related to surgical myectomy and resection of sub-aortic membrane for hypertrophic sub-valvular aortic stenosis. Treatment required pericardiocentesis followed by lymphangiogram with glue embolization of the lymphatic leak. (**Level of Difficulty: Intermediate.**)

## Central Illustration

**Figure:**
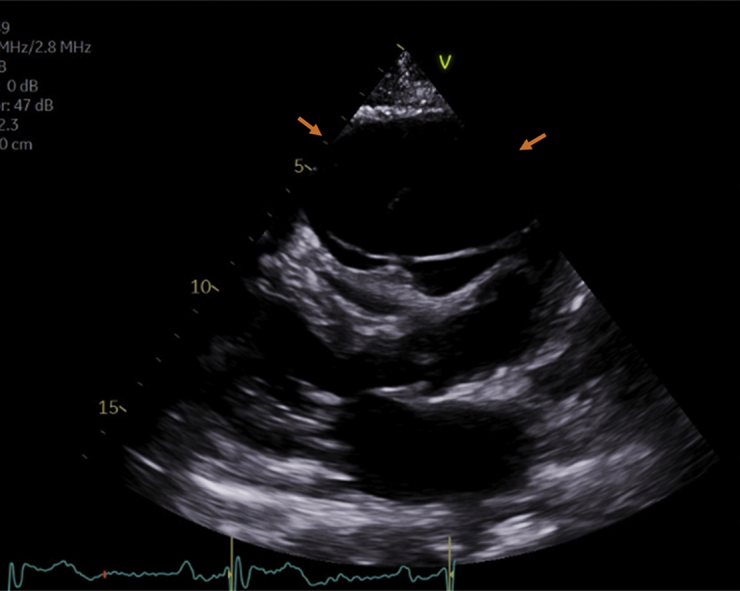

## History of Presenting Illness

A 69-year-old female presented to the clinic with dyspnea and syncope which started a year previously. Her dyspnea was exertional, gradually worsening, relieved upon rest, and associated with chest discomfort. She noticed 3 intermittent syncopal episodes over this period.

**Learning Objectives**

• To understand the pathology, differential diagnosis, and management of recurrent chylopericardium after cardiac surgery.
• To understand the clinical relevance of recurrent chylopericardium.

She denied any orthopnea, paroxysmal nocturnal dyspnea, diaphoresis, palpitations, nausea, vomiting, or abdominal or lower extremities swelling.

## Medical History

Five months previously, she underwent septal myectomy and resection of subaortic membrane for symptomatic left ventricular outflow tract obstruction. Pathology showed mild endocardial fibroelastosis and mild myocyte hypertrophy without disarray or vacuolization. Post-procedure transthoracic echocardiogram (TTE) showed a 2.8-cm large circumferential pericardial effusion (PE) lateral to the left ventricle without any tamponade physiology. Pericardiocentesis drained 900 mL of dark red blood. A pig tail catheter drained an additional 350 mL of pink-colored fluid which then transitioned to milky fluid, another 400 mL. Fluid analysis revealed a total cholesterol level of 52 mg/dl, a triglyceride concentration of 223 mg/dl, and chylomicrons suggestive of chylous PE. No bacterial growth or malignant cells were detected. Because the drain collection was minimal, it was decided to remove the drain after 6 days, and a normal diet was started before the drain removal. Four weeks later, the patient underwent a repeated pericardiocentesis for progressive dyspnea and enlarging PE.

### Physical examination

On presentation, the blood pressure was 130/72 mm Hg, heart rate was 52 beats/min (regular rhythm), respiratory rate was 16 breaths per minute with an oxygen saturation of 97% on room air and a temperature of 98.1°F (36.7°C). Physical examination revealed a pulsatile 3 × 3-cm, round, soft, minimally tender, epigastric mass without skin discoloration. Heart sounds were muffled. Also jugular venous pulse was flat with no pulsus paradoxus.

## Differential Diagnosis

Chylopericardium (recurrent), cholesterol pericarditis, hemorrhagic or purulent PE.

## Investigations

**Figure 1:**
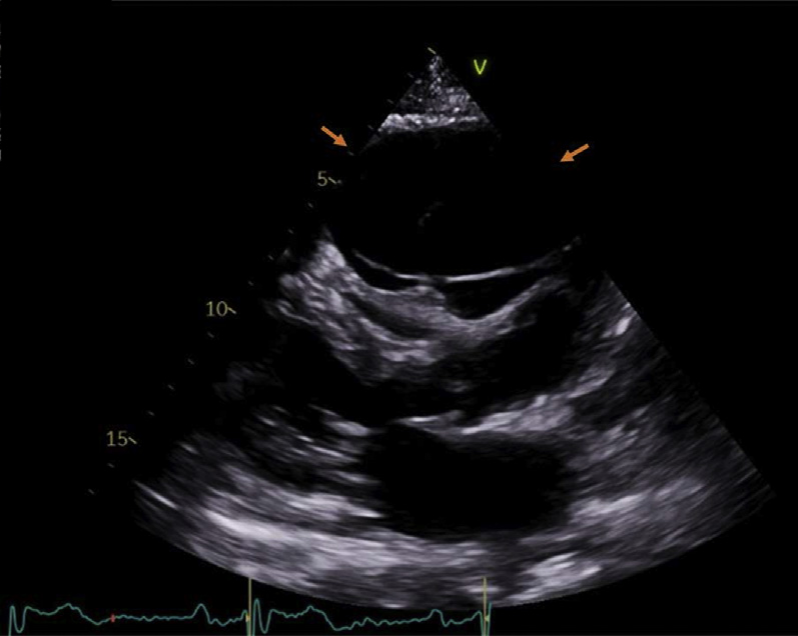
Transthoracic Echocardiogram Shows Anechoic Septated Collection Obstructing the Right Ventricular Outflow Tract

**Figure 2:**
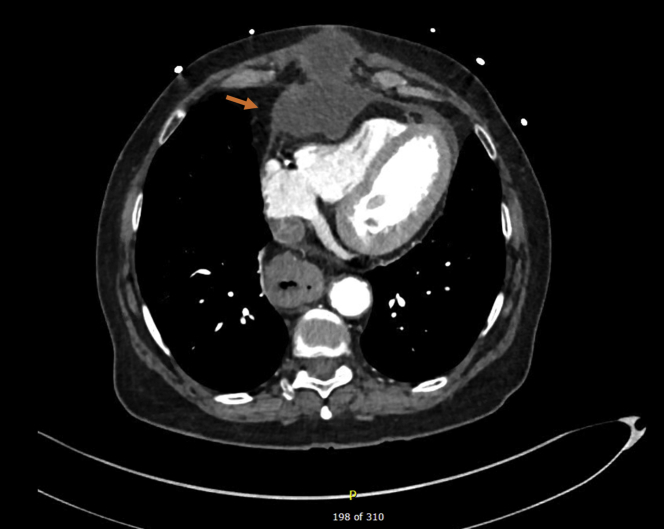
Computed Tomography Shows a Large Pericardial Effusion Anterior and Superior to the Right Ventricle

**Figure 3:**
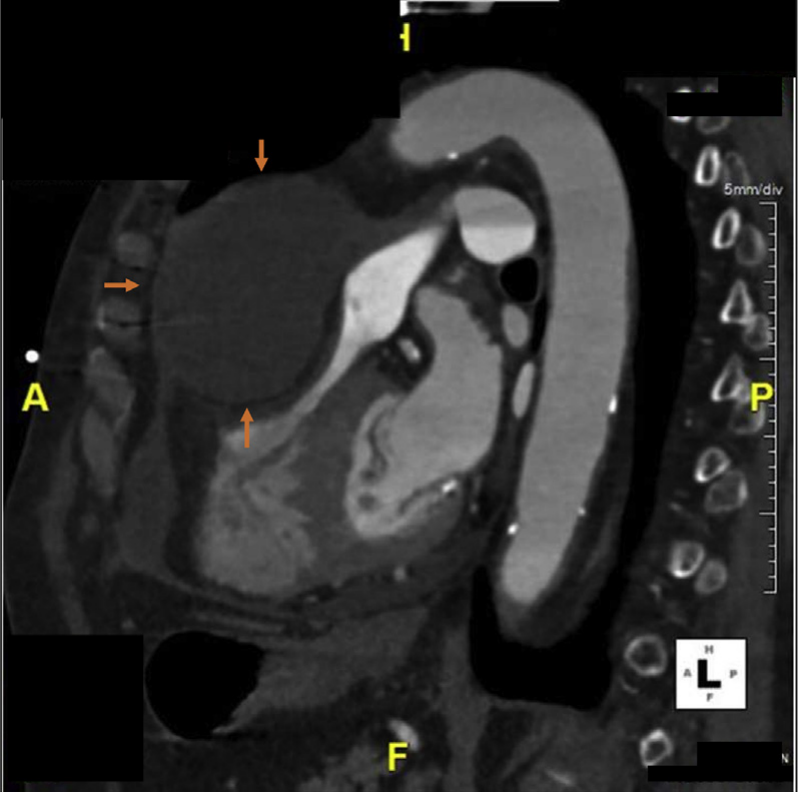
Cardiac Magnetic Resonance Shows a Large Pericardial Effusion Anterior and Superior to Right Ventricle Compressing the Right Ventricular Free Wall

During the patient’s clinic visit, the transthoracic echocardiogram (TTE) showed a large anterior anechoic fluid collection with septations compressing the right ventricular outflow tract (Figure 1). Computed tomography (CT) scans of her chest revealed a large loculated PE (10 × 6 cm) anterior and superior to the right ventricle (Figure 2). Cardiac magnetic resonance (CMR) imaging confirmed the large PE anterior and superior to the right ventricle with septations suggesting partial loculations and compression of the right ventricular free wall most prominent during diastole (Figure 3).

## Management

**Figure 4:**
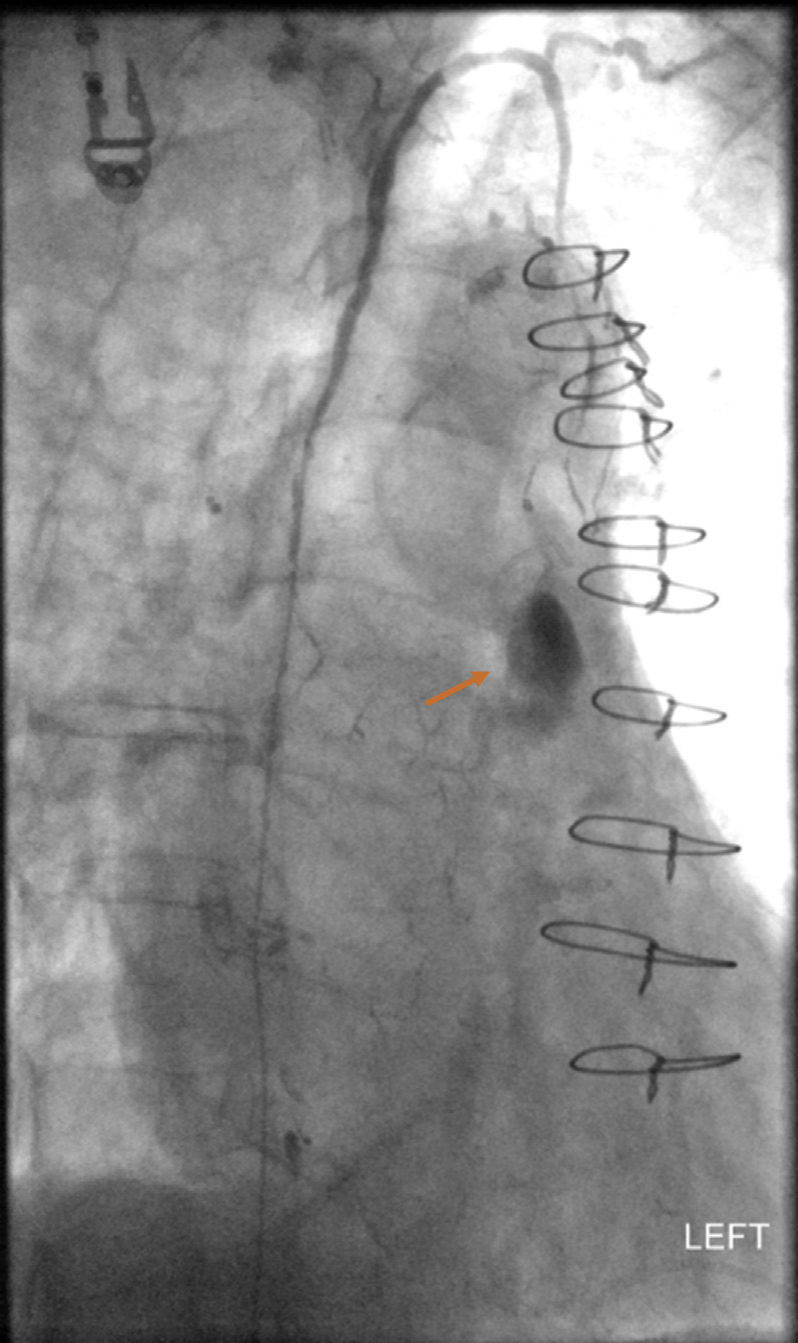
Lymphangiogram Depicting Lymphatic Leak

The patient was admitted to the cardiology service. Pericardiocentesis drained 220 mL of milky fluid. Fluid analysis showed triglycerides of 1,078 mg/dl (>110 mg/dl is suggestive of chylous PE) and chylomicrons (normally absent), 93% lymphocytes and only 3% neutrophils. No bacterial growth or malignant cells were detected. Octreotide was administered but unfortunately was discontinued due to sinus bradycardia. A low-fat diet was started. A pericardial drain was left in place for 5 days, and it drained a total of 940 mL of chylous fluid. Lymphangiography confirmed a lymphatic leak from substernal lymphatics into the anterior mediastinum (Figure 4). A microcatheter was positioned through transabdominal access into the thoracic duct, past the lymphovenous confluence into the anterior mediastinum lymphatics, and successful fluoroscopy- guided glue embolization was performed, followed by removal of the pericardial drain. Repeated TTE showed 1.2-cm small anterior PE adjacent to the right ventricle.

## Follow-Up

At 4 weeks, a telemedicine clinic visit was conducted. She reported reduced dyspnea without any new syncopal episodes.

## Discussion

Chylopericardium (CPE) is a rare and atypical disease entity of the pericardial disease spectrum. Prior cardiac surgery, radiation exposure, malignancy, pancreatitis, and infection are the primary predisposing factors (1). Chyle is the normal content of thoracic duct and lacteals. Many branches of lymphatic fluid from pre-tracheal lymph nodes, pericardial and thymic tissue drain into the thoracic duct. The proposed pathophysiology of development of CPE includes strain on the thoracic duct during manipulation of the cardiac tissue, accompanying lymphatics or development of connections between a lymphatics and pericardial sac (2). CPE after cardiac surgery during median sternotomy is an uncommon condition as the thoracic duct is not in the operative field (3).

Initial diagnostic workup of CPE includes TTE, which shows an echo-free space between the epicardium and parietal pericardium throughout systole and diastole (4). A chest CT with fluid attenuation of −60 to −80 Hounsfield units is highly suggestive of CPE (4). Pericardial fluid analysis when triglyceride levels are >500 mg/dl, a cholesterol-to-triglyceride ratio of <1, Sudan 111 staining, negative fluid cultures, and lymphocyte predominance confirm its diagnosis (1). Multimodality imaging options helpful for the diagnosis are summarized in Table 1 (4). Our patient had a triglyceride level of 1,078 mg/dl with detection of chylomicrons and presence of lymphocytes. Other differential diagnoses of milky appearance of pericardial fluid include cholesterol pericarditis or a purulent PE. Cholesterol pericarditis is diagnosed by characteristic cholesterol crystals and lower triglyceride concentrations, which was not seen in this case (5). Purulent PE, usually bacterial or tubercular in origin, has a high neutrophil count in the fluid with positive cultures. The present case demonstrated 93% lymphocytic predominance with 3% neutrophils in the pericardial fluid with negative cultures.

**Table 1:** Multimodality Imaging Findings in Evaluation of Chylous Pericardial Effusion (4)

Echocardiography	CT Scan	CMR	Lymphangiography
• First-line imaging to detect effusion and tamponade physiology. • Echocardiography-free space between both the pericardial layers throughout the cardiac cycle. • Used to assess the hemodynamics of pericardial effusion.	• Can characterize the type of effusion-pericardial effusion with low attenuation values (−60 to −80 Hounsfield units) characterizes chylopericardium; <10 Hounsfield units suggests transudative effusion; 20 to 60 Hounsfield units can point toward purulent, malignant, or myxomatous effusion. • Can identify complex/loculated effusion. • Can quantify and localize the effusion.	• Can identify effusion as small as 30 mL. • Superior to CT scan in quantifying and localizing effusion. • Tissue can be characterized based on signal intensity. • Can differentiate effusion fluid from pericardial thickening	• Evaluates thoracic duct anatomy. • Can identify the abnormal connection between pericardial cavity and lymphatic system. • Can be combined with CT scan, with and without contrast enhancement.

CMR = cardiac magnetic resonance; CT = computed tomography.

## Management

Management of CPE can be conservative or surgical depending on the severity of symptoms, the underlying cause, or the presence or absence of cardiac tamponade. Conservative management includes medium chain triglyceride or low-fat diet (diminished lymph flow and intralymphatic pressure), and pericardial drainage if needed (1). Octreotide is also used, which reduces chyle production and thoracic duct flow rate (6). European Society of Cardiology (ESC) 2015 guidelines (Class IIa and Level of Evidence: C recommendations) suggest surgical intervention of radical pericardiectomy and treatment of underlying cause when conservative management fails (7).

Pericardiocentesis or pericardial window is required in large or hemodynamically unstable PE (4,7). According to ESC 2015 guidelines, CT with and without contrast enhancement or combined with lymphangiography/lymphoscintigraphy can be used to identify the thoracic duct disruption (7). After identification of the site of leakage, the thoracic duct is embolized proximally (8). Lymphangiography diagnosed the lymphatic leak in our patient further requiring glue embolization without any complications.

Although previous surgery and intervention for valvular heart disease have been associated with its occurrence (9), this case could suggest that myectomy for hypertrophic subaortic stenosis could also be a potential cause. A high clinical suspicion for recurrent CPE is needed during the follow-up of these patients. Gradual or rapid development of clinical symptoms could limit the patient’s daily activities necessitating its timely diagnosis and treatment.

## Conclusions

In summary, recurrent CPE is a rare phenomenon, which can recur most commonly as a sequelae of cardiac surgery. Imaging modalities such as TTE, chest CT, CMR, and lymphangiography serve as important tools for diagnosis and can aid in management. Multidisciplinary care coordination with cardiologists and cardiothoracic surgeons for pericardial drainage and interventional radiology for thoracic duct embolization may be needed for successful outcomes. This case highlights the need for strong clinical suspicion and the role of multimodality imaging to guide management of this rare condition.

## Funding Support and Author Disclosures

Dr. Klein has received research funding from Kiniksa Pharmaceuticals, Ltd; and he has served on scientific advisory boards for Kiniksa Pharmaceuticals, Ltd, Swedish Orphan Biovitrum AB, and Pfizer. The other authors have reported that they have no relationships relevant to the contents of this paper to disclose.
